# Butein Inhibits Angiogenesis of Human Endothelial Progenitor Cells via the Translation Dependent Signaling Pathway

**DOI:** 10.1155/2013/943187

**Published:** 2013-06-06

**Authors:** Ching-Hu Chung, Chien-Hsin Chang, Shiou-Sheng Chen, Hsueh-Hsiao Wang, Juei-Yu Yen, Che-Jen Hsiao, Nan-Lin Wu, Yen-Ling Chen, Tur-Fu Huang, Po-Chuan Wang, Hung-I Yeh, Shih-Wei Wang

**Affiliations:** ^1^Department of Pharmacology, Tzu Chi University, Hualien 970, Taiwan; ^2^Institute of Pharmacology, College of Medicine, National Taiwan University, Taipei 100, Taiwan; ^3^Division of Urology, Taipei City Hospital, Renai Branch, Taipei 106, Taiwan; ^4^Department of Urology, National Yang-Ming University School of Medicine, Taipei 112, Taiwan; ^5^Department of Medicine, Mackay Medical College, New Taipei City 252, Taiwan; ^6^School of Respiratory Therapy, College of Medicine, Taipei Medical University, Taipei 110, Taiwan; ^7^Department of Dermatology, Mackay Memorial Hospital, Hsinchu 300, Taiwan; ^8^Department of Fragrance and Cosmetic Science, College of Pharmacy, Kaohsiung Medical University, Kaohsiung 807, Taiwan; ^9^Department of Gastroenterology, Mackay Memorial Hospital, Hsinchu 300, Taiwan; ^10^Department of Internal Medicine, Mackay Memorial Hospital, Taipei 104, Taiwan

## Abstract

Compelling evidence indicates that bone marrow-derived endothelial progenitor cells (EPCs) can contribute to postnatal neovascularization and tumor angiogenesis. EPCs have been shown to play a “catalytic” role in metastatic progression by mediating the angiogenic switch. Understanding the pharmacological functions and molecular targets of natural products is critical for drug development. Butein, a natural chalcone derivative, has been reported to exert potent anticancer activity. However, the antiangiogenic activity of butein has not been addressed. In this study, we found that butein inhibited serum- and vascular endothelial growth factor- (VEGF-) induced cell proliferation, migration, and tube formation of human EPCs in a concentration dependent manner without cytotoxic effect. Furthermore, butein markedly abrogated VEGF-induced vessels sprouting from aortic rings and suppressed microvessel formation in the Matrigel implant assay *in vivo*. In addition, butein concentration-dependently repressed the phosphorylation of Akt, mTOR, and the major downstream effectors, p70S6K, 4E-BP1, and eIF4E in EPCs. Taken together, our results demonstrate for the first time that butein exhibits the antiangiogenic effect both *in vitro* and *in vivo* by targeting the translational machinery. Butein is a promising angiogenesis inhibitor with the potential for treatment of cancer and other angiogenesis-related diseases.

## 1. Introduction

Angiogenesis plays a critical role in physiological conditions such as embryonic development, reproduction, tissue repair, and bone remodeling. In contrast, angiogenesis is an important process for tumor progression and various inflammatory diseases [1]. Angiogenesis is the result of complex effect on cell-cell and cell-matrix interactions. This process mainly involves endothelial cells proliferation, migration, tube formation, and extracellular matrix (ECM) degradation [2]. Vascular endothelial growth factor (VEGF) is the most potent angiogenic factor, which is primarily secreted by cancer cells to mediate tumor angiogenesis via binding to VEGF receptor (VEGF-R). Therefore, targeting VEGF/VEGF-R axis to block angiogenesis is currently an attractive therapeutic approach for cancer treatment [3, 4].

Circulating endothelial progenitor cells (EPCs) have been shown to play important roles in maintaining vascular integrity and facilitating tissue repair. Circulating EPCs are mobilized from the bone marrow into the bloodstream and induce neovascularization during tissue ischemia [5, 6]. Emerging evidence suggests that EPCs have the ability to self-renew, circulate, home to tumor sites, and differentiate into mature endothelial cells that contribute to angiogenesis and vasculogenesis during the growth and metastatic spread of tumors [7]. Tumor-derived cytokines, such as VEGF, regulate the mobilization of EPCs, which subsequently contribute to tumor angiogenesis and the growth of certain tumors [8]. EPCs reportedly mediate the progression of micrometastasis and subsequently promote tumor macrometastasis, as critical regulators of the angiogenic switch. These findings establish the role of EPCs in tumor angiogenesis and metastasis and support that selective targeting of EPCs may merit investigation for antiangiogenic treatment of metastatic cancer [9, 10].

Translational control has a crucial impact on cancer development and progression, directing both mRNA translation and protein synthesis that regulate tumor cell proliferation, transformation, angiogenesis, and metastasis [11]. Numerous molecular signals have been demonstrated to regulate translational signaling pathways. Earlier studies have shown that Akt and MAPK pathways regulate protein translation through its downstream mammalian target of rapamycin (mTOR) [12, 13]. In eukaryotes, 95–97% of total cellular mRNA translation is via cap-dependent pathway, and the others are through cap-independent pathway [14]. The best-understood roles of mTOR in mammalian cells are tightly associated with the control of cap-dependent mRNA translation. mTOR conducts this translational pathway through phosphorylation of two downstream effectors, the 70 kDa ribosomal protein S6 kinase (p70S6K) and eukaryotic initiation factor 4E binding protein 1 (4E-BP1) [15, 16]. P70 S6 kinase phosphorylates the 40S ribosomal subunit protein S6 and is involved in translational control of 5′ oligopyrimidine tract mRNAs. Unphosphorylated 4E-BP1 is a translational inhibitor that binds to eukaryotic initiation factor 4E (eIF4E) to repress translation initiation. The activation of mTOR leads to hierarchical phosphorylation of 4E-BP1, dislodging 4E-BP1 from eIF4E, and subsequently increasing cap-dependent translation [11]. mTOR-mediated translational signaling is important for cellular proliferation and growth in endothelial cells and various tumor cells [17, 18]. Deregulation of mTOR signaling is frequently associated with tumor growth and angiogenesis [16, 19]. Thus, mTOR signaling pathway is central to translational regulation and is a novel target for cancer therapeutics.

Butein (3,4,2′,4′-tetrahydroxychalcone), a type of chalcone derivative, has been identified from numerous plants including the heartwood of *Dalbergia odorifera *(namely, Jiangxiang in Chinese), the stem bark of cashews (*Semecarpus anacardium*), and the traditional Chinese and Tibetan medicinal herbs such as *Caragana jubata* and *Rhus verniciflua* Stokes. Previous reports have demonstrated that butein has various pharmacological effects, such as antioxidant and anti-inflammatory activities [20, 21], elicitation of endothelium-dependent vasodilation [22], antirestenosis effect [23], and anticancer effects in a variety of human cancer cells [24–29]. Several chalcones have been reported to exhibit antiangiogenic activity via blocking VEGF-induced angiogenesis [30, 31]. However, the antiangiogenesis property of butein is mostly unknown. In this study, we investigated the antiangiogenic activity of butein in both *in vitro* and* in vivo* assays and further elucidated its mechanism of action in human EPCs.

## 2. Materials and Methods

### 2.1. Materials

3,4,2′,4′-Tetrahydroxychalcone (butein) was purchased from Extrasynthese Corporation (Genay, France). 3-[4,5-Dimethylthiazol-2-yl]-2,5-diphenyltetrazolium bromide (MTT), crystal violet, and other chemicals were purchased from Sigma (St Louis, MO). MV2 complete medium was purchased from PromoCell (Heidelberg, Germany). Defined fetal bovine serum (FBS) and all cultured reagents were purchased from HyClone (Logan, UT). Recombinant human VEGF was purchased from R&D Systems (Minneapolis, MN). Lactate dehydrogenase (LDH) assay reagents were purchased from Promega (Madison, WI). Antibodies to phospho-Akt (Ser473), Akt, phospho-mTOR (Ser2448), and phospho-eIF4E (Ser209) were purchased from Epitomics (Burlingame, CA). Antibodies to phospho-ERK1/2 (Thr202/Tyr204), ERK1/2, phospho-p70S6K (Thr389), phospho-4E-BP-1 (Thr37/46), and GAPDH were purchased from Cell Signaling Technologies (Boston, MA).

### 2.2. Isolation and Cultivation of EPCs

Ethical approval was granted by the Institutional Review Board of Mackay Medical College, New Taipei City, Taiwan (reference number: P1000002). Informed consent was obtained from healthy donors before the collection of peripheral blood (80 mL). The peripheral blood mononuclear cells (PBMCs) were fractionated from other blood components by centrifugation on Ficoll-Paque plus (Amersham Biosciences, Uppsala, Sweden) according to the manufacturer's instructions. CD34-positive progenitor cells were obtained from the isolated PBMCs using CD34 MicroBead Kit and MACS Cell Separation System (Miltenyi Biotec, Bergisch Gladbach, Germany). The isolation and maintenance of CD34-positive EPCs were performed as described previously [32, 33]. Briefly, human CD34-positive EPCs were maintained and propagated in MV2 complete medium consisting of MV2 basal medium and growth supplement, supplied 20% FBS. Cells were seeded onto 1% gelatin-coated plasticware and cultured in humidified air containing 5% CO2 at 37°C for further treatment. Characterization of EPCs was confirmed by UEA-1 binding, and surface marker staining of CD34, KDR, and CD31. (see Supplementary Figure 1 in Supplementary Material available online at http://dx.doi.org/10.1155/2013/943187). Experiments were conducted on EPCs between passages 8 and 12.

### 2.3. Cell Proliferation Assay

For crystal violet assay, EPCs (5 × 10^3^ cells/well) were seeded onto 96-well plates. After 24 h incubation, the culture medium was removed and cells were incubated with fresh MV2 complete medium containing 10% FBS for 72 h in the absence or presence of butein. Then, cell proliferation was determined by crystal violet staining, which was performed by staining with 0.5% crystal violet in 20% methanol. The dye was subsequently eluted in the solution containing 0.1 M sodium citrate and 75% ethanol, and absorbance was measured at 540 nm with ELISA-reader.

For MTT colorimetric assay, EPCs (5 × 10^3^ cells/well) were incubated in 96-well plates and starved with MV2 complete medium for 16 h. Then, the culture medium was removed, and cells were incubated with fresh MV2 complete medium containing VEGF (20 ng/mL) for 48 h in the absence or presence of butein. Cells were incubated with MTT (0.5 mg/mL) for 2 h. Formazan crystal was lysed by dimethyl sulfoxide (DMSO), and absorbance was measured at 550 nm with ELISA-reader.

### 2.4. Cytotoxicity Assay

EPCs were seeded onto 96-well plates in a density of 5 × 10^3^ cells per well and starved with MV2 complete medium for 16 h. Then, cells were treated with MV2 complete medium containing VEGF (20 ng/mL) for 48 h in the absence or presence of butein. The percentage of LDH release was calculated from the ratio of LDH activity in the medium to LDH activity in the cell lysate.

### 2.5. Cell Migration Assay

Cell migration assay was performed using Transwell chambers with 8.0 *μ*m pore size (Coring, Coring, NY). EPCs (5 × 10^4^ cells/well) were seeded onto the upper chamber with MV2 complete medium and then incubated in the bottom chamber with MV2 complete medium containing 10% FBS or VEGF (20 ng/mL) with the indicated concentrations of butein. After 16 h of treatment, cells on the upper side of the filters were mechanically removed, and those which migrated on the lower side were fixed with 4% formaldehyde then stained with 0.5% crystal violet for 10 min. Cell migration was quantified by counting the number of stained cells in 10 random fields with the inverted phase contrast microscope and photographed.

### 2.6. Capillary Tube Formation Assay

Matrigel (BD Biosciences, Bedford, MA), which was used to promote the differentiation of EPCs into a capillary tube-like structure, was added to 48-well plates. The Matrigel-coated 48-well plates were incubated at 37°C for 30 min to allow for polymerization. After gel formation, EPCs (6 × 10^4^ cells) were seeded per well on the layer of polymerized Matrigel in MV2 complete medium containing 10% FBS or VEGF (20 ng/mL) with the indicated concentrations of butein, followed by incubation for 10–16 h at 37°C. Photomicrographs of capillary tube formation were taken with the inverted phase contrast microscope. Tube formation was quantified by measuring the long axis of each tube in 3 random fields per well by using Image-Pro Plus software.

### 2.7. Aortic Ring Sprouting Assay

Assay was performed as previously described with modification [34]. Aortas were harvested from 8- to 10-week-old Sprague-Dawley rats. Following a complete washing, the aortas were cut into 1 mm ring segments. The aortic rings were placed in the 48-well plates which were precoated with 130 *μ*L Matrigel and polymerized at 37°C. The wells were subsequently overlaid with another 50 *μ*L Matrigel for sealing. VEGF (20 ng/mL) with or without butein was then added to the well. The cultured medium was changed every 3 days. Sprouting endothelial cells were observed and photographed on day 8. The area of sprouting vessels was measured quantitatively by Image-Pro Plus software.

### 2.8. Directed *In Vivo* Angiogenesis Assay (DIVAA)

DIVAA was performed as described previously [35]. Briefly, sterile, surgical silicone tubes were filled at 4°C with matrigel containing VEGF with or without butein. Therefore, the dorsal haunches of the anesthetized mice (8- to 10-week-old female C57BL/6 mice) were shaved and sterile prepped. A 5 mm cutaneous incision was made, and a 10 mm deep subcutaneous pocket was created with a sterile hemostat. DIVAA tubes were incubated at 37°C for 1 hour to allow gel formation and then implanted into the dorsal flank of mice. After 15 days, DIVAA tubes were taken and photographed. Neovessels were quantified by measuring the hemoglobin of the plug with the Drabkin method and Drabkin reagent kit 525 (Sigma). The animals were maintained on a 12-hour light/dark cycle under controlled temperature (20 ± 1°C) and humidity (55 ± 5%). Animals were given continuous access to food and water. All procedures involving animal experiment were approved by the Institutional Animal Care and Use Committee at College of Medicine, Tzu Chi University.

### 2.9. Western Blot Analysis

Cells were lysed with lysis buffer as described previously [36]. Cell homogenates were diluted with loading buffer and boiled for 5 min for detecting phosphorylation and protein expression. Total protein was determined and equal amounts of protein were separated by 8–12% SDS-PAGE and immunoblotted with specific primary antibodies. Horseradish peroxidase-conjugated secondary antibodies (Santa Cruz Biotechnology, Santa Cruz, CA) were used, and the signal was detected using an enhanced chemiluminescence detection kit (Amersham, Buckinghamshire, UK).

### 2.10. Statistical Analysis

Data are presented as the mean ± SEM for the indicated number of separate experiment. Statistical analyses of data were performed with one-way ANOVA followed by Student's *t*-test, and *P* values less than 0.05 were considered significant.

## 3. Results

### 3.1. Butein Inhibits Cell Proliferation of EPCs without Cytotoxicity

**Figure 1:**
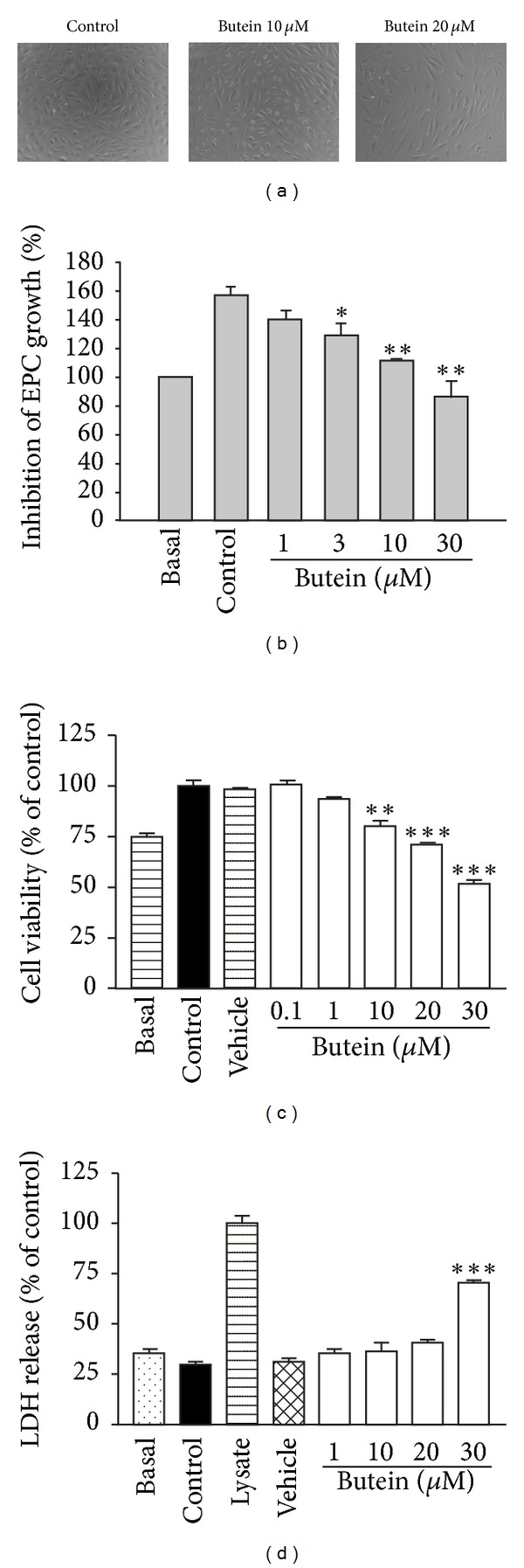
Effect of butein on cell proliferation and cytotoxicity of EPCs. Cells were seeded into a 96-well plate at 5000 cells per well overnight for attachment. After 72 h incubation with the indicated concentrations of butein in MV2 complete medium containing 10% FBS, cell morphology was detected by inverted phase contrast microscope (a), and cell proliferation was determined using crystal violet assay (b). Serum-starved cells were stimulated with or without VEGF (20 ng/mL) in the absence or presence of various concentrations of butein, and the cell growth and cytotoxicity were determined using MTT assay and LDH assay, respectively (c and d). Data are expressed as mean ± SEM of five independent experiments. _ _*P < 0.05, _ _**P < 0.01,  and _ _***P < 0.001 compared with the control group.

To assess the antiangiogenic activity of butein, we first evaluated the influence of butein on cell proliferation of EPCs. The results showed that butein induced the cytostatic effect in human EPCs (Figure 1(a)). Furthermore, butein suppressed serum- or VEGF-induced cell proliferation of EPCs in a concentration-dependent manner (Figures 1(b) and 1(c)). In order to investigate whether the effect of butein was due to its cytotoxicity, LDH assay was performed. There is no significant increase in LDH release when the concentration is lower than 20 *μ*M. However, treatment of higher concentration of butein (30 *μ*M) caused a significant LDH release (Figure 1(d)). Therefore, the concentration of butein used in the following research is less than 20 *μ*M.

### 3.2. Butein Inhibits Cell Migration of EPCs

**Figure 2:**
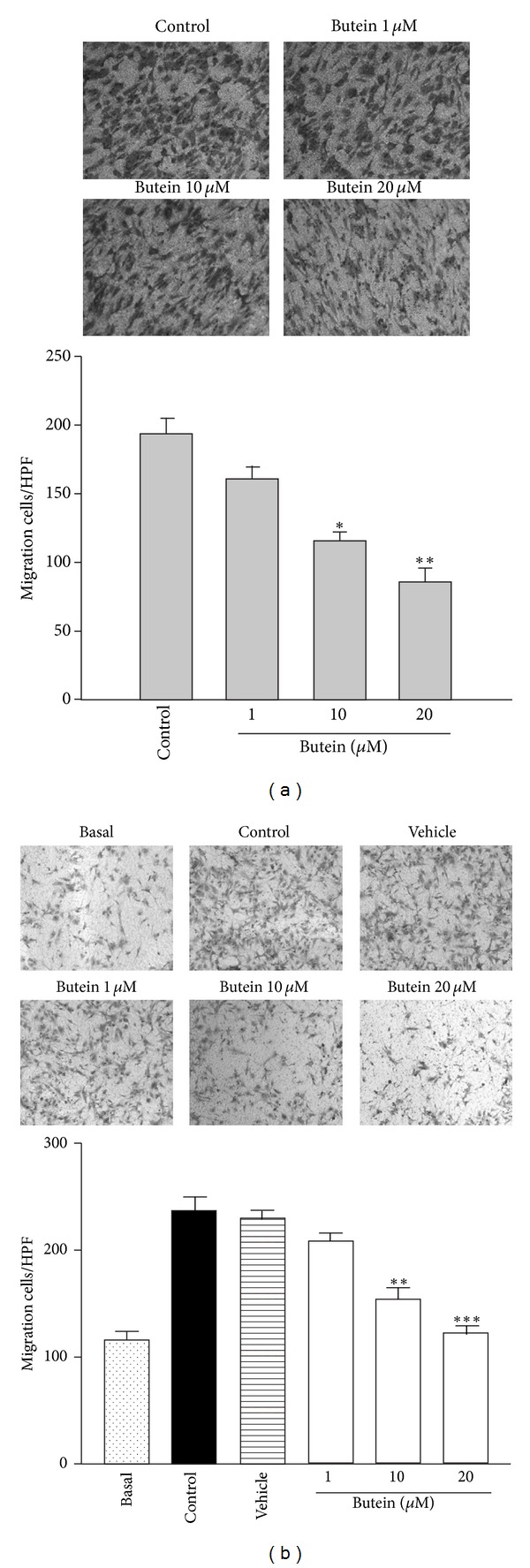
Effect of butein on cell migration of EPCs. Cells were seeded onto the upper chamber consisting of 8 mm pore-size filters and then treated with the indicated concentrations of butein in MV2 complete medium containing 10% FBS (a) or 20 ng/mL VEGF (b) as a chemoattractant in the lower chamber. Cells that invaded the filter were counted as mean ± SEM of five independent experiments. _ _*P < 0.05, _ _**P < 0.0,  and _ _***P < 0.001 compared with the control group.

The migration of EPCs is an important step in angiogenesis. We investigated the chemotactic migration with Transwell chambers to examine the effect of butein on EPC migration toward serum or VEGF. As shown in Figure 2, serum and VEGF induced EPCs migration after 16 hr treatment. Butein significantly attenuated serum- and VEGF-induced cell migration.

### 3.3. Butein Inhibits Tube Formation of EPCs

**Figure 3:**
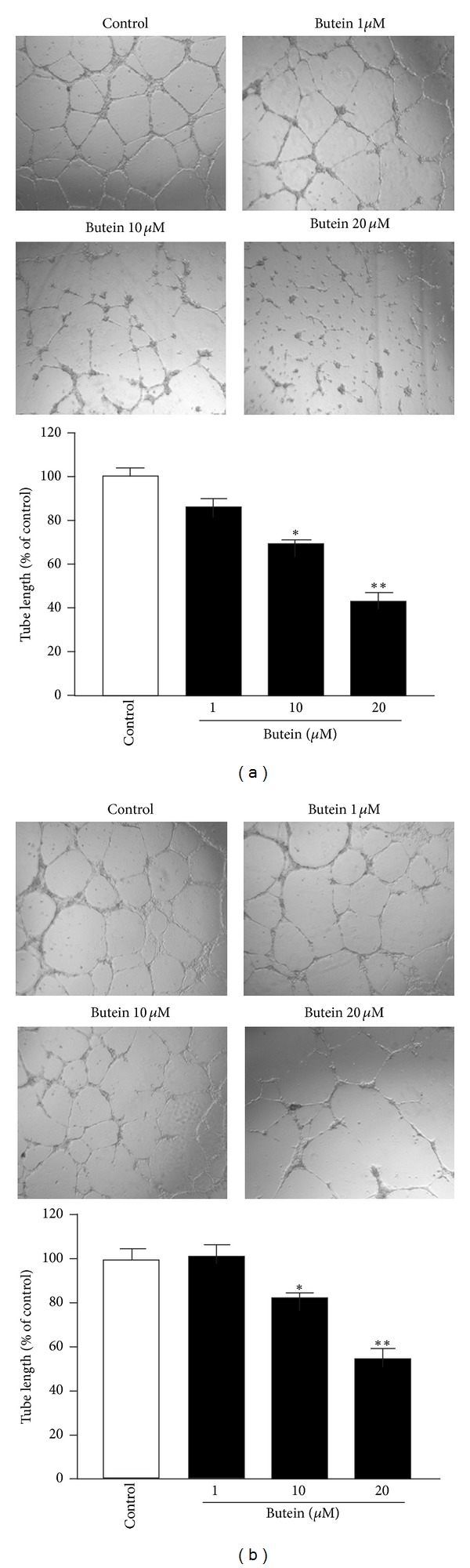
Effect of butein on tube formation of EPCs. Cells were plated on Matrigel-coated plates in the presence of MV2 complete medium containing 10% FBS (a) or 20 ng/mL VEGF (b) with various concentrations of butein, and tubular morphogenesis was recorded by the inverted phase contrast microscope. Tube formation was quantified by measuring the length of tubes in three random fields per well with the use of Image-Pro Plus and was calculated against DMSO control. Data are expressed as mean ± SEM of four independent experiments. _ _*P < 0.05, _ _**P < 0.01 compared with the control group.

In order to study the effect on EPCs differentiation and formation of capillary-like structure, we tested whether butein inhibited tube formation on Matrigel. The capillary tube-like structure was facilitated by angiogenic factors such as serum and VEGF. After 10 hours of treatment, butein exhibited the promising inhibitory effect on serum- or VEGF-induced tube formation (Figure 3). These results demonstrate that butein has the ability to block *in vitro* angiogenesis of EPCs.

### 3.4. Butein Inhibits Angiogenesis *Ex Vivo *and* In Vivo*

**Figure 4:**
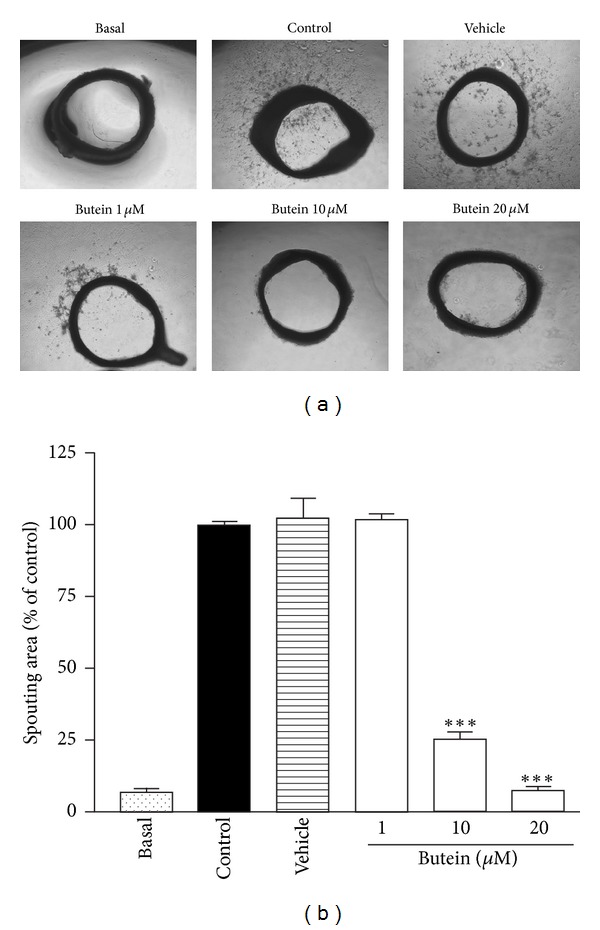
Effect of butein on VEGF-induced angiogenesis *ex vivo*. Aortas in Matrigel were treated with or without VEGF (20 ng/mL) in the absence or presence of various concentrations of butein. After 8 days, aortic rings were photographed. Experiments were repeated four times, and a representative result is shown. Data are expressed as mean ± SEM of four independent experiments. _ _***P < 0.001 compared with the control group.

**Figure 5:**
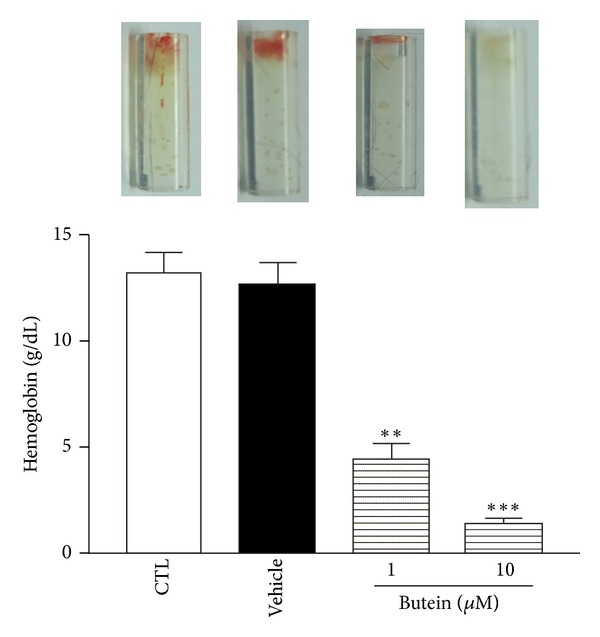
Effect of butein on VEGF-induced angiogenesis* in vivo*. Matrigel containing VEGF (200 ng/mL) with or without butein in sterile, surgical silicone tubes were subcutaneously placed into C57BL/6 mice. After 15 days, DIVAA tubes was taken and photographed. Hemoglobin was measured as an indication of blood vessel formation, using the Drabkin method. Data are presented as mean ± SEM of at least 3 mice per group. _ _**P < 0.01, _ _***P < 0.001  compared with control group.

We further performed the aortic ring sprouting assay to evaluate *ex vivo* antiangiogenic effect of butein. As shown in Figure 4, VEGF apparently stimulated the vessels sprouting of the rat aortic ring, whereas butein significantly decreased the sprouting of VEGF-induced vessels in a concentration-dependent manner. To determine whether butein is capable of blocking angiogenesis* in vivo*, we used an *in vivo* mouse DIVAA model. An aliquot of growth factor-reduced Matrigel containing VEGF was filled into a sterile, surgical silicone tubing and then inoculated subcutaneously into mouse. New vessels were induced by VEGF and formed a capillary network in the DIVAA tubes. As shown in Figure 5, butein demonstrated the inhibitory effect of microvessel in growth into the DIVAA tubes. Quantification of angiogenesis by hemoglobin content revealed that butein significantly inhibited the angiogenic response in a dose-dependent manner. These results suggest that butein strongly blocks VEGF-induced angiogenesis* ex vivo* and *in vivo*.

### 3.5. Butein Inhibits the Translational Pathway in EPCs

**Figure 6:**
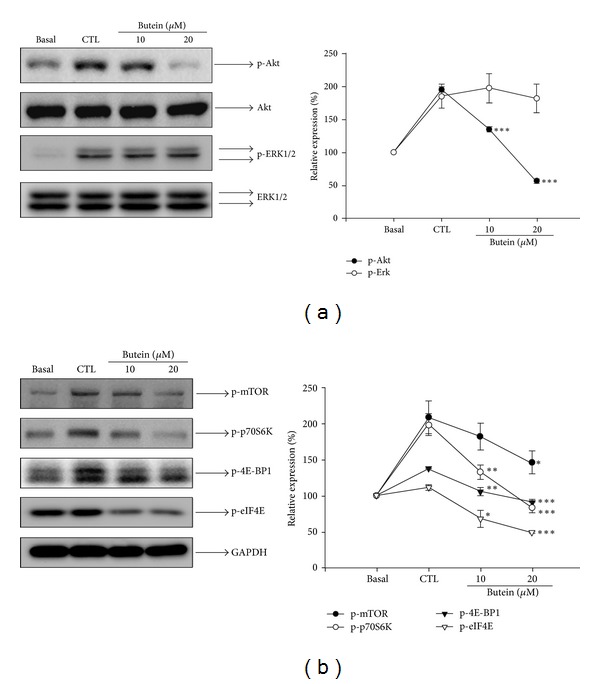
Effects of butein on the phosphorylation of Akt, ERK1/2, and translational regulatory proteins. Quiescent EPCs were treated with or without MV2 complete medium containing 10% FBS in the absence (control) or presence of butein (10 and 20 *μ*M) for 15 min. Cells were harvested and lysed for the detection of p-Akt and p-ERK1/2 (a), p-mTOR, p-p70S6K, p-4E-BP1, and p-elf4E (b) by Western blot analysis. The quantitative densitometry of the relative level of protein was performed with Image-Pro Plus. Data are expressed as mean ± SEM of five independent experiments. _ _*P < 0.05, _ _**P < 0.01,  and _ _***P < 0.001 compared with the control (CTL) group.

Both phosphatidylinositol-3-kinase (PI3K)/Akt and extracellular signal-related kinase 1/2 (ERK1/2) signaling pathway play important roles in cell proliferation, survival, migration, and angiogenesis [18, 37]. Therefore, we investigated these molecular mechanisms underlying the effects of butein-mediated intracellular signal transduction in EPCs. As shown in Figure 6(a), phosphorylation of Akt and ERK1/2 were markedly increased after serum stimulation. Butein dramatically inhibited the phosphorylation of Akt in a concentration-dependent manner but did not significantly suppress the phosphorylation of ERK1/2 in EPCs. The important downstream effector of PI3K/Akt pathway termed mTOR is recognized to regulate the translational process through increased phosphorylation of p70S6K and 4E-BP1. In addition, mTOR and p70S6K signal have been indicated to regulate various biological functions of endothelial cells (ECs) for angiogenesis [17, 19]. Thus, we explored whether butein modulated these signaling pathways in EPCs. As shown in Figure 6(b), serum induced a significant increase in the phosphorylation of mTOR, p70S6K, 4E-BP1, and eIF4E. We found the phosphorylation of mTOR and the major downstream targets, p70S6K, 4E-BP1, and eIF4E, were significantly suppressed in EPCs by butein treatment. These results suggest that butein may inhibit angiogenesis probably through the inhibition of translational process.

## 4. Discussion

The development of new blood vessels from preexisting ones is generally referred to angiogenesis, which plays a critical role in tumor growth and metastasis. In recent years, anti-angiogenic agents have become attractive strategy for cancer treatment and provide some additional benefits, including fewer side effects and lower dosage of chemotherapy [3, 38]. Accumulating evidence indicates that tumor angiogenesis is also supported by the mobilization and functional incorporation of other cells such as endothelial progenitor cells (EPCs) [6]. Recently, EPCs have been proposed to improve early tumor growth and late tumor metastasis by intervening with the angiogenic switch; EPCs promote tumor neovessel formation through the production of angiogenic cytokines during tumor progression [8, 39]. Furthermore, several studies demonstrate that certain chemotherapy drugs can trigger circulating EPCs mobilization and subsequent tumor homing [10, 40]. Therefore, EPC-targeting therapies may be the promising strategy to block angiogenesis-mediated tumor growth. In this study, we used EPCs to investigate the effect of butein on angiogenesis. We found that butein inhibited serum- and VEGF-induced proliferation in a concentration-dependent manner (Figure 1). Tumor cells release chemoattractants, such as VEGF, into microenvironment and stimulate cell migration and angiogenic effects to form capillaries. We showed that butein reduced the ability of EPCs to migrate towards serum and VEGF (Figure 2). Butein markedly and concentration-dependently inhibited serum- and VEGF-induced capillary-like tubular formation of EPCs (Figure 3). Notably, we demonstrated the potent *ex vivo* and *in vivo* anti-angiogenic effect of butein, suggesting that butein may be a potential angiogenesis inhibitor (Figures 4 and 5).

Butein that is derived from numerous traditional herbal medicines displayed promising anticancer activities against a broad spectrum of human cancer cells via different mechanisms. Butein inhibited migration and invasion through the ERK1/2 and NF-*Κ*b signaling pathways in bladder cancer cells [25]. Butein suppressed invasion and CXCR4 expression in breast and pancreatic cancer cells [26]. Butein was found to be an aromatase inhibitor with growth inhibitory effect in breast cancer cells [29]. In addition, butein inhibited cell growth and induced apoptosis in melanoma, leukemia, and hepatocellular carcinoma cells [24, 27, 28]. The concentration of butein for its anticancer effects varies from 5 to 200 *μ*M depending on its mechanism, time treatment, and tumor cell type. Mostly, butein induced the growth inhibitory and apoptotic effects in cancer cells at a higher concentration around 25–200 *μ*M. In the present study, the concentration of butein (<20 *μ*M) did not induce LDH release of EPCs, indicating that this antiproliferation effect of butein was not due to its cytotoxicity (Figures 1(c) and 1(d)). Butein at a concentration of 10 *μ*M significantly inhibited angiogenesis of human EPCs and human umbilical vein endothelial cells (HUVECs) (see Supplementary Figure 2). The growth inhibitory action of butein at low concentration (1–10 *μ*M) is considered to be a more specific effect on endothelial cells that are stimulated by angiogenic growth factors. Taken together, we suggest that low concentration of butein exhibits novel anti-angiogenic activity and may contribute to suppress tumor growth and metastasis *in vivo*.

Importantly, our study is the first to show the effect of butein on AKT/mTOR-mediated translational pathway. Previous evidence implies that Akt and Erk signaling is the key mediator for vasculogenic functions, such as proliferation, survival, migration, and differentiation, which are activated by a variety of stimuli in endothelial cells and EPCs [37, 41]. Several studies showed that the inhibition of Akt and ERK1/2 signaling in tumor vasculature resulted in vessel reduction and tumor growth suppression [42–44]. We found that butein significantly inhibited serum-induced Akt phosphorylation in a concentration-dependent manner, but not Erk1/2 phosphorylation in EPCs (Figure 6(a)). These results indicated that butein may suppress angiogenesis via inhibition of the Akt-dependent pathway. Akt signaling plays an important role in the activation of mTOR pathway, which has been indicated to regulate various biological functions of ECs for angiogenesis [13, 17]. Recent study also demonstrates that mTOR is involved in the control of cell growth in EPCs [45]. The mTOR signaling pathway is central to translational regulation and is a pivotal target in cancer therapeutics. Through activation of its downstream p70S6K and hyperphosphorylation of 4E-BP1, AKT/mTOR pathway coordinates and conducts cap-dependent mRNA translation in not only cell proliferation and growth, but also a crucial step leading to angiogenesis in the neoplastic and nonneoplastic processes [11, 46]. Our results showed that butein dramatically inhibited the mTOR signaling cascade, including mTOR, p70S6K, 4E-BP1, and elf4E in EPCs (Figure 6(b)). Thus, we suggest that anti-angiogenic activity of butein may be through the suppression of translation signaling pathway.

In conclusion, this study discovers a novel mechanism by which butein impairs angiogenesis *in vitro* and *in vivo*. We demonstrate that butein inhibits EPCs proliferation, migration, and tube formation by targeting the AKT/mTOR translation dependent signaling pathway. Since the contribution of EPCs during tumor angiogenesis is important for the initiation and promotion of tumor neovessel formation, our data provide evidence for butein that could be a potential candidate of angiogenesis inhibitor. Based on the findings herein, we suggest that butein is a promising natural product worthy of further development for the treatment of human cancer and other angiogenesis-related diseases.

## Supplementary Material

Figure S1. Characterization of human CD34-positive endothelial progenitor cells. (A) Representative flow cytometry analysis showed that EPCs were positive for CD34, KDR, and CD31. (B) The endothelial cell property was examined by UEA-1 staining. Corresponding negative isotype controls are shown in black.
Figure S2. Effects of Butein on tube formation and migration of HUVECs. (A) HUVECs (1.2x10^5^ cells/well) were treated with the indicated concentration of butein for 16 h in the presence of VEGF (20 ng/ml), and tubular morphogenesis was recorded by microscope. Tube formation was quantified by measuring the length of tubes in three random fields per well with the use of Image-Pro Plus. (B) HUVECs (5x10^4^ cells/well) were seeded onto the upper chamber, then treated with the indicated concentrations of butein for 16 h in the presence of VEGF (20 ng/ml) as a chemoattractant in the lower chamber. Cells that migrated to the underside of the filter membrane were stained, photographed, and quantified by microscope. Data are expressed as mean ± S.E.M. of three independent experiments. *∗∗∗p* < *0.001* compared with the control group.
