# Supplementary material for: Butein Inhibits Angiogenesis of Human Endothelial Progenitor Cells via the Translation Dependent Signaling Pathway
Source: Evid Based Complement Alternat Med. 2013 Jun 6;2013:943187. doi: 10.1155/2013/943187 (PMC3690248; doi:10.1155/2013/943187)
Supplement: Supplementary file 1 — Figure S1. Characterization of human CD34-positive endothelial progenitor cells. (A) Representative flow cytometry analysis showed that EPCs were positive for CD34, KDR, and CD31. (B) The endothelial cell property was examined by UEA-1 staining. Corresponding negative isotype controls are shown in black. Figure S2. Effects of Butein on tube formation and migration of HUVECs. (A) HUVECs (1.2x10^5^ cells/well) were treated with the indicated concentration of butein for 16 h in the presence of VEGF (20 ng/ml), and tubular morphogenesis was recorded by microscope. Tube formation was quantified by measuring the length of tubes in three random fields per well with the use of Image-Pro Plus. (B) HUVECs (5x10^4^ cells/well) were seeded onto the upper chamber, then treated with the indicated concentrations of butein for 16 h in the presence of VEGF (20 ng/ml) as a chemoattractant in the lower chamber. Cells that migrated to the underside of the filter membrane were stained, photographed, and quantified by microscope. Data are expressed as mean ± S.E.M. of three independent experiments. ∗∗∗p < 0.001 compared with the control group. [file 943187.f1.pdf]

Supplementary Methods

Flow cytometry analysis

After magnetic labeling and separation, isolated CD34-positive progenitor cells were maintained with MV2 complete medium. Human CD34-positive EPCs cells were collected at passage 8 and applied to flow cytometry analysis. 2×10^5 cells were incubated with antibodies specific to CD31, CD34 (both from BD Pharmingen), VEGF receptor-2 (KDR; R&D Systems), and *Ulex europaeus* agglutinin-1 lectin (UEA-1; Sigma). Isotype-identical antibodies were used as controls. After 30 minutes of incubation under 4℃ in dark, cells were washed twice and resuspended in HBSS buffer immediately followed by analysis using FACScan flow cytometer (BD Biosciences).

HUVECs culture

Human umbilical vein endothelial cells (HUVECs) were purchased from Bioresource Collection and Research Center (Hsinchu, Taiwan) and maintained in M199 containing 20% fetal bovine serum (FBS), 30 μg/ml endothelial cell growth supplements (ECGs), 4 mM L-glutamine, 100 U/ml penicillin and 100 μg/ml streptomycin. Cells were incubated at 37 °C in a humidified atmosphere of 5% CO2. Then, HUVECs were used for tube formation and migration assay, respectively.

Supplementary Figures

Supplementary figure 1

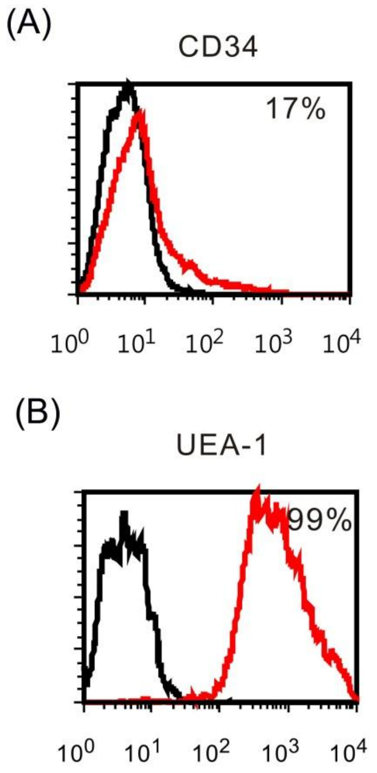

Figure S1. Characterization of human CD34-positive endothelial progenitor cells.
(A) Representative flow cytometry analysis showed that EPCs were positive for CD34, KDR, and CD31. (B) The endothelial cell property was examined by UEA-1 staining. Corresponding negative isotype controls are shown in black.

Supplementary figure 2

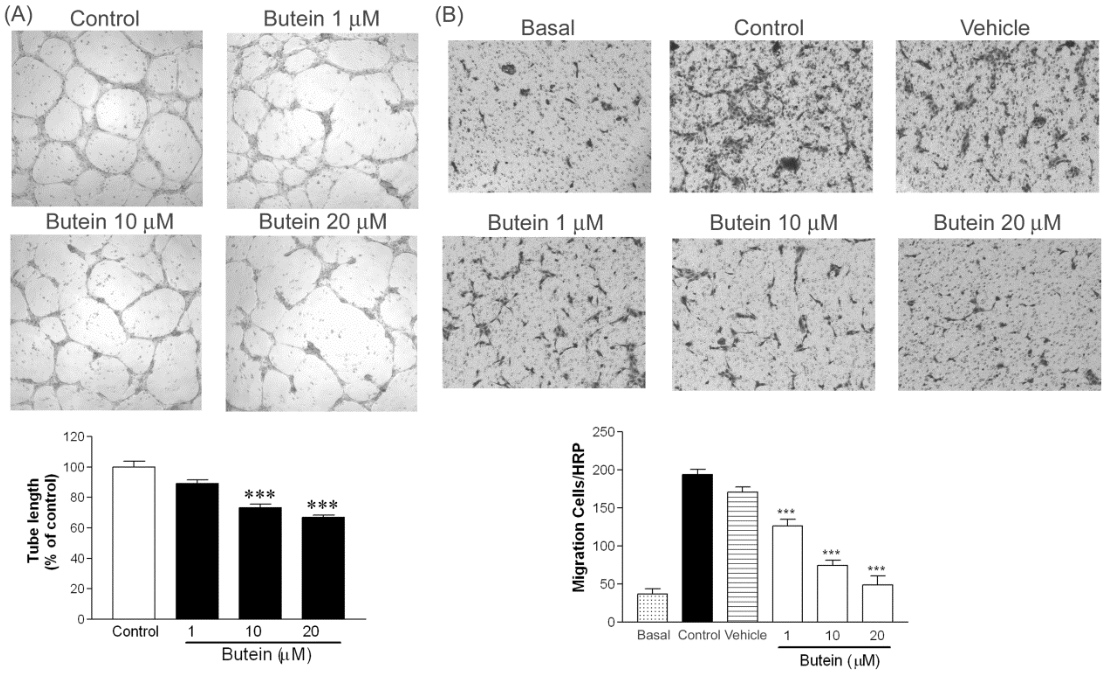

Figure S2. Effects of Butein on tube formation and migration of HUVECs. (A) HUVECs (1.2×10^5 cells/well) were treated with the indicated concentration of butein for 16 h in the presence of VEGF (20 ng/ml), and tubular morphogenesis was recorded by microscope. Tube formation was quantified by measuring the length of tubes in three random fields per well with the use of Image-Pro Plus. (B) HUVECs (5×10^4 cells/well) were seeded onto the upper chamber, then treated with the indicated concentrations of butein for 16 h in the presence of VEGF (20 ng/ml) as a chemoattractant in the lower chamber. Cells that migrated to the underside of the filter membrane were stained, photographed, and quantified by microscope. Data are expressed as mean ± S.E.M. of three independent experiments. ***$p < 0.001$ compared with the control group.
